# Preferred oriented cation configurations in high pressure phases IV and V of methylammonium lead iodide perovskite

**DOI:** 10.1038/s41598-020-77852-y

**Published:** 2020-12-03

**Authors:** Wiwittawin Sukmas, Vichawan Sakulsupich, Prutthipong Tsuppayakorn-aek, Udomsilp Pinsook, Teerachote Pakornchote, Rakchat Klinkla, Thiti Bovornratanaraks

**Affiliations:** 1 Extreme Conditions Physics Research Laboratory (ECPRL), Physics of Energy Materials Research Unit (PEMRU), Department of Physics, Faculty of Science, Chulalongkorn University, Bangkok, 10330 Thailand; 2 Thailand Center of Excellence in Physics, Ministry of Higher Education, Science, Research and Innovation, 328 Si Ayutthaya Road, Bangkok, 10400 Thailand

**Keywords:** Materials science, Materials for energy and catalysis, Energy science and technology, Energy harvesting, Physics, Condensed-matter physics, Electronic properties and materials, Phase transitions and critical phenomena

## Abstract

A microscopic viewpoint of structure and dipolar configurations in hybrid organic–inorganic perovskites is crucial to understanding their stability and phase transitions. The necessity of incorporating dispersion interactions in the state-of-the-art density functional theory for the $CH_3NH_3PbI_3$ perovskite (MAPI) is demonstrated in this work. Some of the vdW methods were selected to evaluate the corresponding energetics properties of the cubic MAPI with various azimuthally rotated MA organic cation orientations. The highest energy barrier obtained from PBEsol reaches 18.6 meV/MA-ion, which is equivalent to 216 K, the temperature above which the MA cations randomly reorient. Energy profiles calculated by vdW incorporated functionals, on the other hand, exhibit various distinct patterns. The well-developed vdW-DF-cx functional was selected, thanks to its competence, to evaluate the total energies of different MA dipolar configurations in $2\times 2\times 2$ cubic supercell of MAPI under pressures. The centrosymmetric arrangement of the MA cations that provide zero total dipole moment configuration results in the lowest energy state profiles under pressure, while the non-centrosymmetric scheme displays a unique behaviour. Despite being overall unpolarised, the latter calculated with PBEsol leads to a rigid shift of energy from the profile obtained from the dispersive vdW-DF-cx functional. It is noteworthy that the energy profile responsible for the maximum polarised configuration nevertheless takes the second place in total energy under pressure.

## Introduction

The emergence of hybrid organic-inorganic perovskites (HOIPs) has attracted tremendous worldwide attention due to their highly potential applications, for instance, in optoelectronic and photovoltaic technology^1–5^. The structural and dynamical nature of such systems and the presence of ferroelectric domains that reduce the rate of electron–hole pair recombination were suggested^6^, as well as the interaction between the embedded molecular cations and the inorganic framework^7^ as interpreted through energy-landscape analyses^8–10^, to impact upon the photovoltaic performance of these materials. Methylammonium lead iodide perovskite (MAPI) and Formamidinium lead iodide (FAPI) photovoltaic cells, being the most familiar archetypal HOIPs, are comparatively inexpensive and also easy to assemble^11^. Recently, MAPI solar cell has gained the efficiency up to more than 22%^12–14^, yet by exploiting a mixture of *Cs* and *I*/*Br*, the *Cs*/*FA*/*Br*/*I*-mixed HOIP was demonstrated the feasibility of achieving more than 25%-efficient tandem cells^15^. The innovative and low-cost synthetic design delivers hopeful prospect for the commercialisation of the perovskite solar cells, even though there are still many challenges waiting to be resolved, e.g. reducing non-radiative recombination and increasing conductivity of device layers^16^.

One major problem is that HOIPs are highly unstable. They are susceptible to the attacks of humidity, ultraviolet light, and thermal stress^4,17–19^. The average lifespan of the most investigated perovskite solar cells is in the order of weeks to only several months^20^. Even the most stable cell reported was guaranteed to last only for a year under controlled conditions^21^, still immature in a world dominated by the well-established silicon solar cells. As for the theoretical investigations, especially for the density functional theory (DFT), the stability issues have reportedly been addressed in the context of dispersive interaction, e.g. the importance of van der Waals (vdW) interaction between the $PbI_6$ octahedra and the methylammonium (MA) cation evidenced in electronic property was acknowledged by dispersion-corrected DFT^22^, as well as the computational predictions regarding structural parameters that substantiate the vdW interactions among the halide atoms and hydrogen bonding^23,24^. On the other hand, many efforts have continuously been made to tackle the stability problems of this class of materials. A set of alternative protonated cations were substituted in place of the MA and formamidinium (FA) cations so as to enhance the structural stability of the HOIPs by strengthening hydrogen bondings^25^. Not only was a mixture of *Cs*/*MA*/*FA* cations incorporated with an attempt to achieve high efficiency perovskite solar cells that are structurally stable under operational conditions^15,26^, the critical role played by grain boundaries were also elucidated to facilitate ion migration, leading to a rearranged ion distribution that eventually enhances the stability of the ion-mixed perovskite^27^.

**Figure 1:**
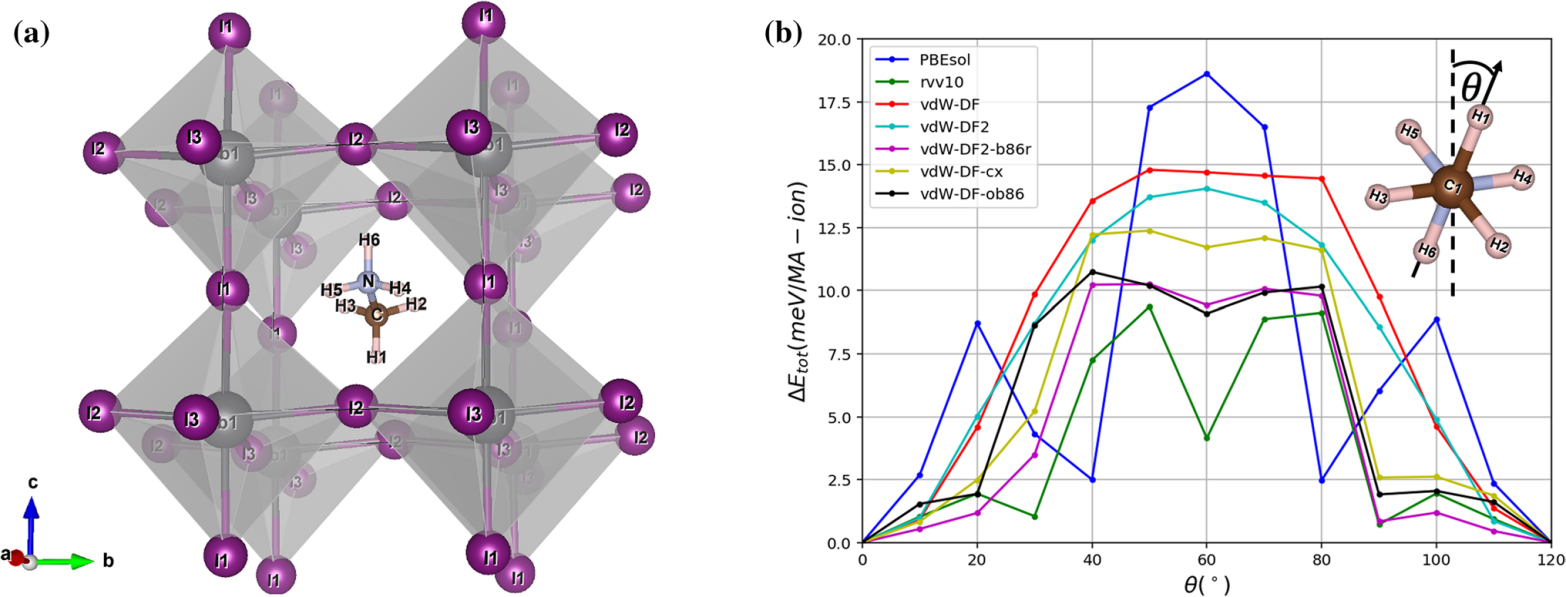
Details of energy barrier evaluation: (**a**) The cubic structure of MAPI with a lattice parameter of 6.317 Å  as input structure embedding an organic MA cation, with labelled atoms; (**b**) Total energy per cation profiles calculated by different functionals, taking the lowest total energy as a reference^28^; Inset depicts an applied anticlockwise rotation about the *a*-axis for the MA cation.

Apart from stability problem, researches that focus on the electronic property, namely, improvement of electronic band gaps, were conducted with the aim of having it approached the Shockley–Queisser (SQ) limit or the maximum theoretical efficiency of a solar cell^29^. Moreover, the extent of angular distortions of the $SnI_4$ inorganic cage was also found to greatly affect the tuning of the band gaps^30^, as well as the orientations of the organic cations^31^ Furthermore, three types of organic cations, i.e. $Cs^+$, MA, and FA, with a respective increase of sizes, were emphasised to be influential in stabilising the crystal structure by means of an enhancement of hydrogen bonding^23,24,32^.

In similar manner, substituting $Br^-$ (larger) with $Cl^-$ (smaller) results in an increase of band gaps, as experimentally and theoretically inspected^33^. More interestingly, irrespective of chemical modification, crystal structures can straightforwardly be morphed, whose changes in atomistic geometry and bonding characteristics are achieved, by pressurisation, as implemented in methylammonium lead halide perovskites^34–36^. Upon an increase of pressure, MAPI was synthesised and subsequently demonstrated to undergo a series of phase transitions^35,37^. At ambient pressure, the material, regardless of the MA cationic orientation, adopts a tetragonal phase II, space group *I*4/*mcm*, structure in the temperature range between 165 K and 321 K^7^ and the pressure range to 0.35 GPa, while it morphs into a cubic phase IV, $Im\bar{3}$, structure at 0.3–2.5 GPa and isostructural cubic phase V, $Im\bar{3}$, above 2.5 GPa^37^. Also, experimental data corroborated the distinction of phases IV and V in terms of molecular volumes, *Pb*–*I* bond lengths, $Pb{-}I{-}Pb$ bond angles, and the energy gaps, which in principle exhibit disruptive jumps during structural phase transitions^7^.

To this end, it is of utmost importance to further investigate the effects of the organic molecular orientations on structural property of phases IV and V in relation to the unambiguously available experimental results^38^ by exploiting the first-principles calculations based on the state-of-the-art density functional theory incorporating vdW interactions. The rigid flips of the MA cation were applied to determine energy profiles accounting for various types of dispersion-corrected versions of vdW interactions, which are believed to be greatly affecting the models of ours. We found that vdW interactions greatly affect the energy barriers of the MA cation embedded in $PbI_6$ when evaluated by different types of functionals. Also, the cationic orientational configurations suggest a relatively stable structure for MAPI under pressures.

**Figure 2:**
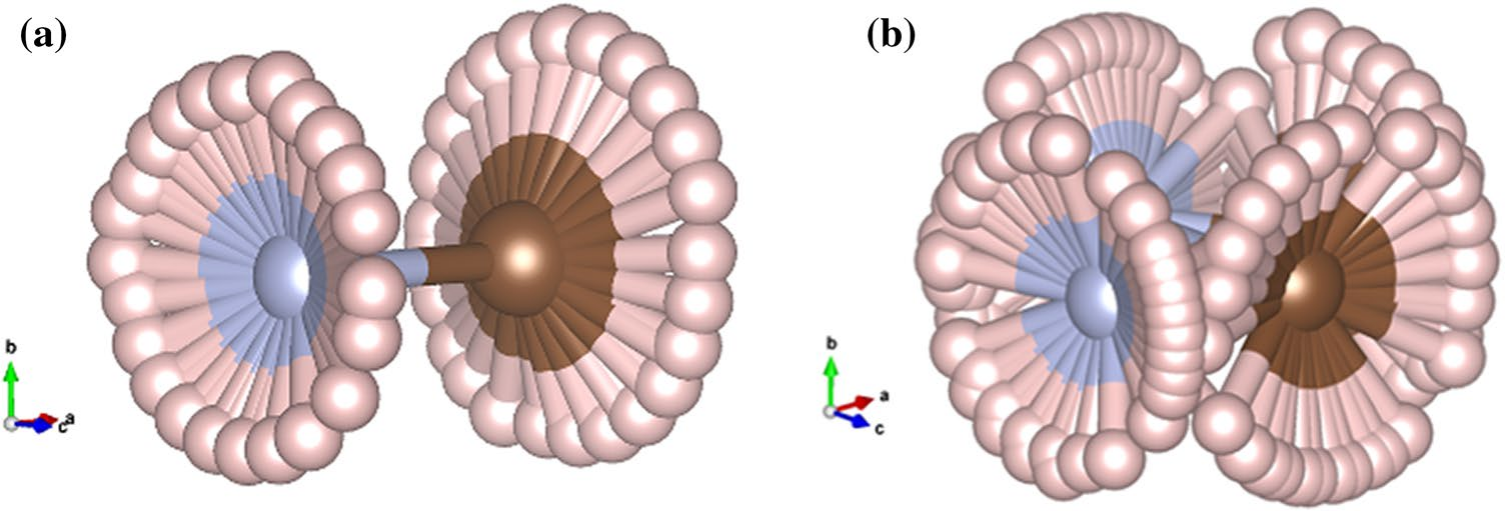
Two types of voids in $Im\bar{3}$ space group. We introduce simplified notations of the structure and the voids. $S_n$ denotes a spherical void; x-D, y-D, and z-D denote dumbbell voids in their respective orientation. The MA cation in $S_n$ (**a**) and x-D void (**b**).

## Results ans discussion

### Energy barriers

As earlier mentioned, the interplay between the octahedral tilts and the MA cationic dynamics still requires a clearer picture. Suggestions were made that the molecular dynamics of the organic MA cation are associated with the rotation about the $C{-}N$ axis that is in average achieved in sub-picosecond time scale^39^. In addition to that, the effect of the organic MA molecular orientations has been proposed to play a decisive role for the structural stability, indirectly affected by vdW interactions, and other physical properties of these hybrid materials^40^. Thus it is the purpose of ours to carry out a careful inspection of the organic–inorganic interaction by the aids of PBEsol and/with different methods of incorporated vdW dispersion, including rVV10^41^, vdW-DF^42,43^, vdW-DF2^44^, vdW-DF2-b86r^45,46^, vdW-DF-cx^47^, vdW-DF-ob86^48^ functionals.

Illustrated in Fig. 1(a) with labelled atoms, the pseudocubic MAPI of space group $Im\bar{3}$ with an average lattice parameter of 6.37 Å, comprising an inorganic framework of $PbI_6$ within which embeds at its centre a dipolar organic cation of $CH_3NH_3^+$ (MA), was initially selected as a starting structure. Due to $C_{3v}$ group or threefold symmetry, the MA cation was then applied a set of rigid flips through an angle $\theta$ [See the inset of Fig. 1(b)] running from $0^\circ$ to $120^\circ$, which were discretised into 13 turning steps with an each $10^\circ$ step size. Also, all calculations were based on the assumption that the octahedral framework responses quickly to the rotations, and henceforth the whole system was fully relaxed under rotational operations.

The results are reported in Fig. 1(b). Along the vertical axis plots angle-dependence total energy difference per cation compared with the lowest one, denoted as $\Delta E_{tot}$ (meV/MA-ion). All profiles appear to be nearly symmetric about $\theta = 60^\circ$ owing to the threefold symmetry of the MA cation (see the inset). Regardless of vdW interaction, PBEsol functional, designed to achieved enhanced equilibrium properties of densely-packed solids^49,50^ and based on a fit of the exchange-correlation energy to that of the surface jellium^51^, blatantly gives a distinct energy profile (blue) from those of van der Waals functionals, namely at $\theta = 60^\circ$ it resembles a highest barrier with $\Delta E_{tot}$ = 18.6 meV/MA-ion and is flanked by minor peaks of $\sim$9 meV/MA-ion at $\theta = 20^\circ$ and $100^\circ$. PBEsol explicitly disregards the dispersive interaction of $H{-}I$ pairs that eventually entails a set of comparatively larger distances (see Fig. S1 in Supplementary Information), resulting in a highest peak at $60^\circ$. With the inclusion of vdW interactions, however, both valleys formed at $\theta = 40^\circ$ and $80^\circ$ happen to be morphed into single broader, putatively Gaussian-like profiles. The presence of $\theta = 40^\circ$- and $80^\circ$-valleys is presumably stemmed from the fact that the positions of $H$-atoms are located just enough in an optimal level of average $H{-}I$ inter-fragment distances. At $\theta =60^\circ$ the peak, prominent in PBEsol, smears out in vdW-DF (red) and vdW-DF2 (cyan), whereas an erosion of the peak becomes more and more evident when vdW-DF-cx (yellow), vdW-DF2-b86r (magenta), vdW-DF-ob86 (black), and rvv10 (green) were performed in succession. A total energy barrier was described as an entity that relates to thermal excitation, i.e. $\Delta E \sim k_BT$, where $\Delta E$ denotes the flipping energy barrier^10^. The highest barrier, in our case, reads 18.6 meV/MA-ion which is responsible for $T \sim 216 K$. This implies that at temperature higher than this, the MA cation is free to randomly reorient within a void.

An overestimation of equilibrium separations and an underestimation of $H$-bond strengths of vdW-DF^42^, relying on the Langreth-Vosko screened exchange^52^ that was subsequently replaced by the large-$N$ asymptote gradient correction^53^, are improved in vdW-DF2^44^ and result in a lower, unstable equilibrium peak of 21 meV/MA-ion (vdW-DF2) in place of the one with 1 meV/MA-ion higher peak at $60^\circ$. Nonetheless, vdW-DF2-b86r reported to improve equilibrium separations and the $H$-bonding in particular^45,46^, over vdW-DF2 gives rise to a stable equilibrium at $60^\circ$ as well as the one evaluated by the vdW-DF-ob86, which is reportedly suitable for hard matters^48^. More interestingly, the clearest of all excavated points at $60^\circ$ is obtained from the revised version of a Vydrov and van Voorhis^54^ named rVV10^41^. Finally, the total energy profile achieved by vdW-DF-cx^47^, the non-empirical functional utilising the unified vdW-DF plasmon–response representation for both correlation and exchange^42^, exhibits not as completely a Gaussian-like profile. Though the concavity originates from $\sim 1$ meV/MA-ion difference in total energy of neighbouring data points, which is smaller than energy convergence threshold set in this work. Thus, we opted for vdW-DF-cx by virtue of its pinpoint accuracy of lattice parameter predictions and $H{-}I$ distances^9,28,47^. Nine pairs of $H{-}I$ bond distances evaluated by two schemes, i.e., PBEsol and vdW-DF-cx, are plotted in Supplementary Fig. S1, and are also discussed in the SM.

### Dipolar configurations under pressures

**Figure 3:**
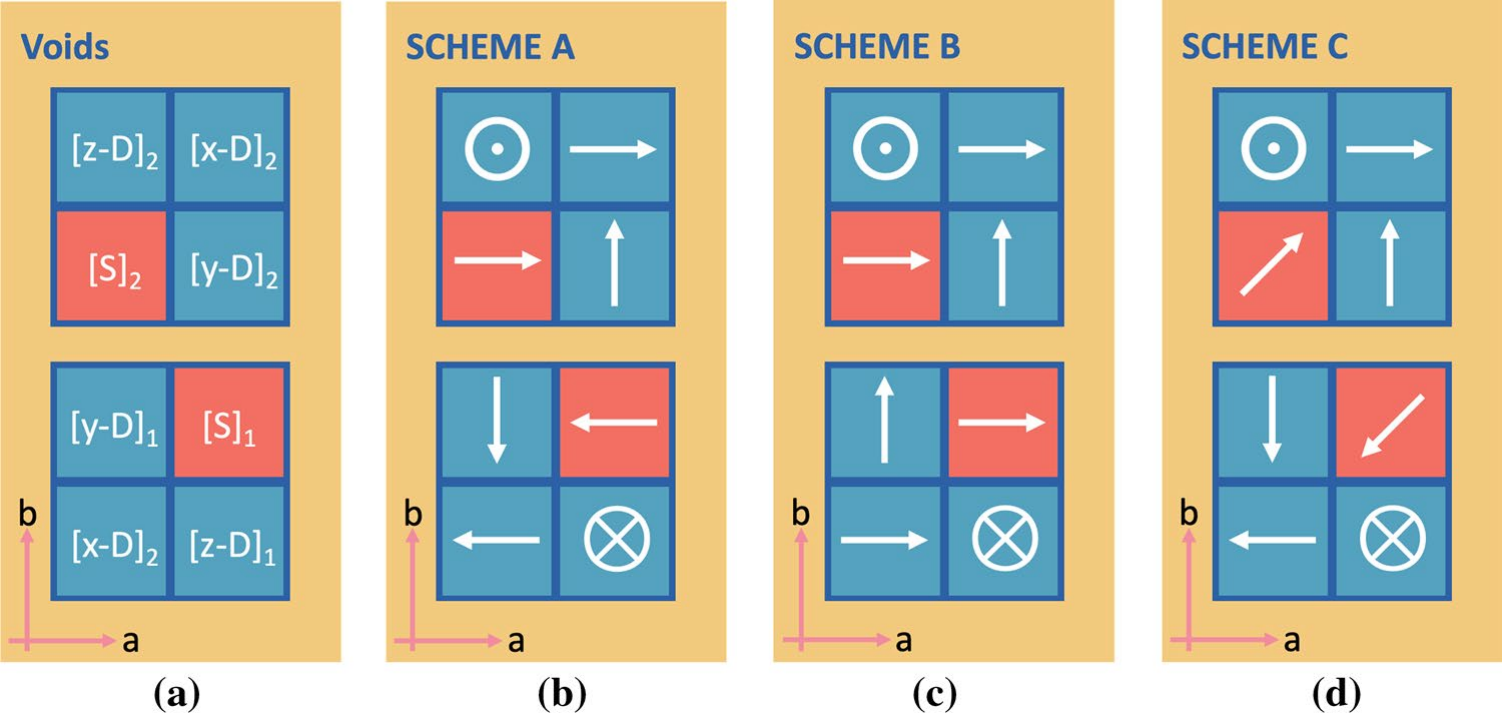
Schematic orientation of the $CH_3NH_3$ (MA) molecules. The diagram plane is in *xy*-plane and the lower diagrams show the bottom layer of the cell. Light red squares are the sphere voids (denoted by $[\mathbf{S} ]_n$), and cyan squares denote the dumbbell-like voids (denoted by $[\mathbf (x, y, z) -\mathbf{D} ]_n$, where $n = 1$ denotes voids in lower layer and $n = 2$ is for upper layer of the simulation cell. Arrows (and symbols) in the diagrams show the orientation of the $CH_3NH_3$ molecule inside each void. The arrow head is *N* and the tail is *C*. Circle $\odot$ means that the arrow is pointing outward from the paper and the cross $\otimes$ means that the arrow pointing into the paper.

Increasing pressure results in a series of phase transitions of MAPI, as clearly demonstrated in Supplementary Fig. S2 by means of enthalpy–pressure relationship and in Supplementary Fig. S3 for the distorted structures. In this section, however, the effect of dipolar orientational configurations under pressure was systematically investigated. As reported by Szafranski *et al.* ^37^, both phases IV and V of MAPI adopt the same space group symmetry, $Im\bar{3}$, of which the unit cell is just a $2\times 2\times 2$ supercell expansion of the cubic phase containing 8 formula units of $MAPbI_3$. Thus there are exactly 8 units of $PbI_6$ octahedra, 8 MA cations, and 8 voids available for the MA cations. Moreover, voids were classified into two types, i.e. elongated or dumbbell-like and spherical voids^37^. We illustrate in Fig. 2 both types of voids in which the MA cation reorients in accordance with the shapes of the given cavity (dumbbell-like and spherical shapes in (a) and (b), respectively). To this end, we investigated the MA molecular orientational behaviours under pressure by first introducing this work’s convention. Illustrated in Fig. 3(a), an initial structure consisting of 8 cubic unit cells of MAPI, where the upper layer (top view of the structure) accounts for $n=2$ and the lower $n=1$. A dumbbell-like void is able to align in three possible configurations, namely along [100] ($[x-D]_n$), [010] ($[y-D]_n$), and [001] ($[z-D]_n$) directions, whereas the spherical void is denoted as $S_n$. The $C-N$ dipole is represented by an arrow where its head corresponds to *N*, and its tail *C*. Thus there are 2 possible orientations in a dumbbell-like void available for the MA cation. However, in the simulation cell, there are two *x*-dumbbells, two *y*-dumbbells, two *z*-dumbbells and two spherical voids. Therefore, there are in total $2^6\times$(possibility of MA spherical rotation)^2^ ways to place the MA inside the voids.

**Figure 4:**
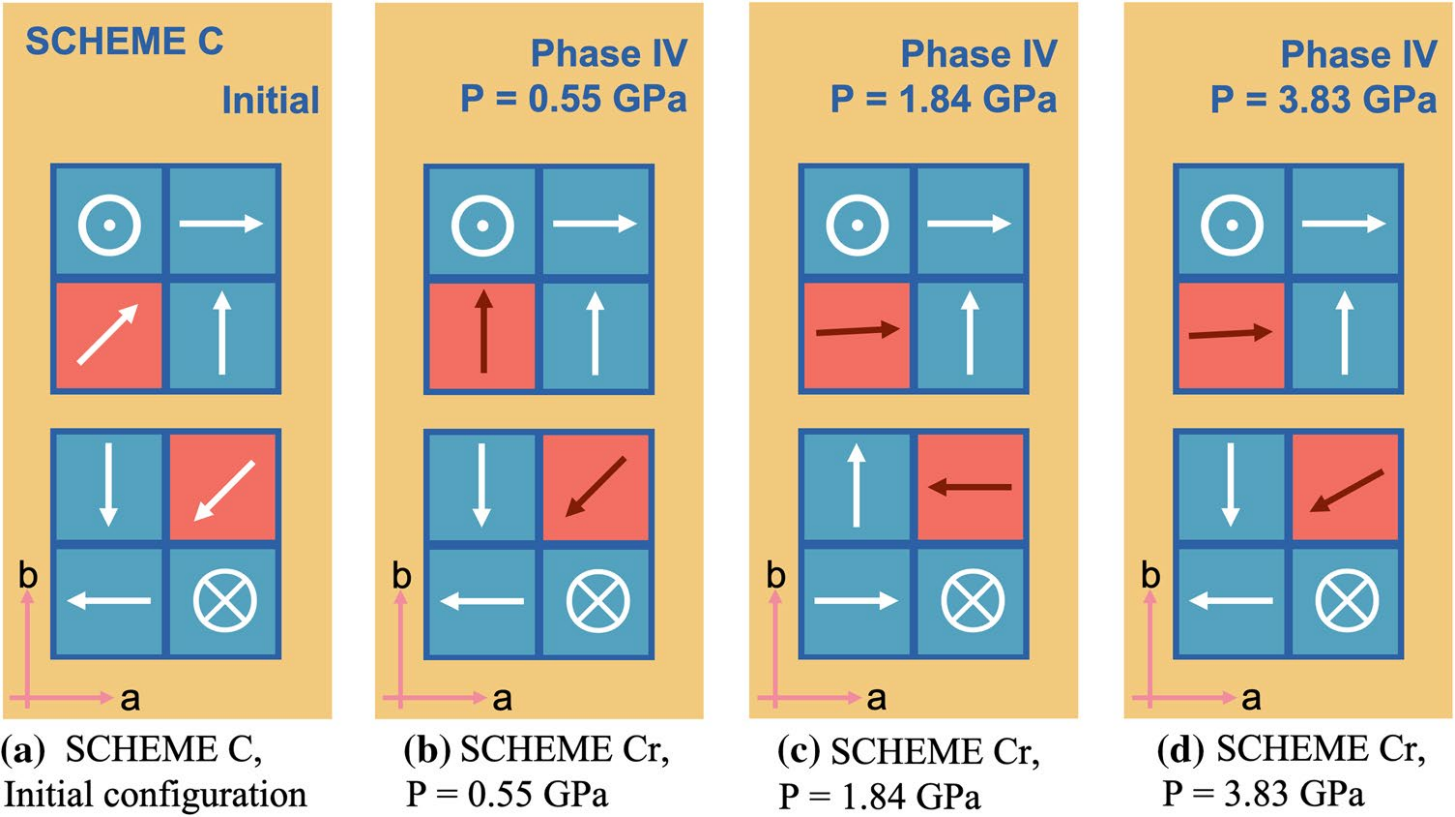
Schematic orientation of MA molecules. By structural optimisation starting from Scheme C in (**a**), the MA evolved into (**b**) and (**c**) at low pressure (0.55–2.5 GPa), and (**d**) at higher pressure (2.7–3.83 GPa).

Amongst a large number of possibilities of how the MA cation can align, we chose to investigate only a number of extreme cases as follows:

- Scheme A wherein gives the zero total dipole moment.
- Scheme B wherein gives the maximum total dipole moment.
- Scheme C wherein also gives the zero total dipole moment.

**Figure 5:**
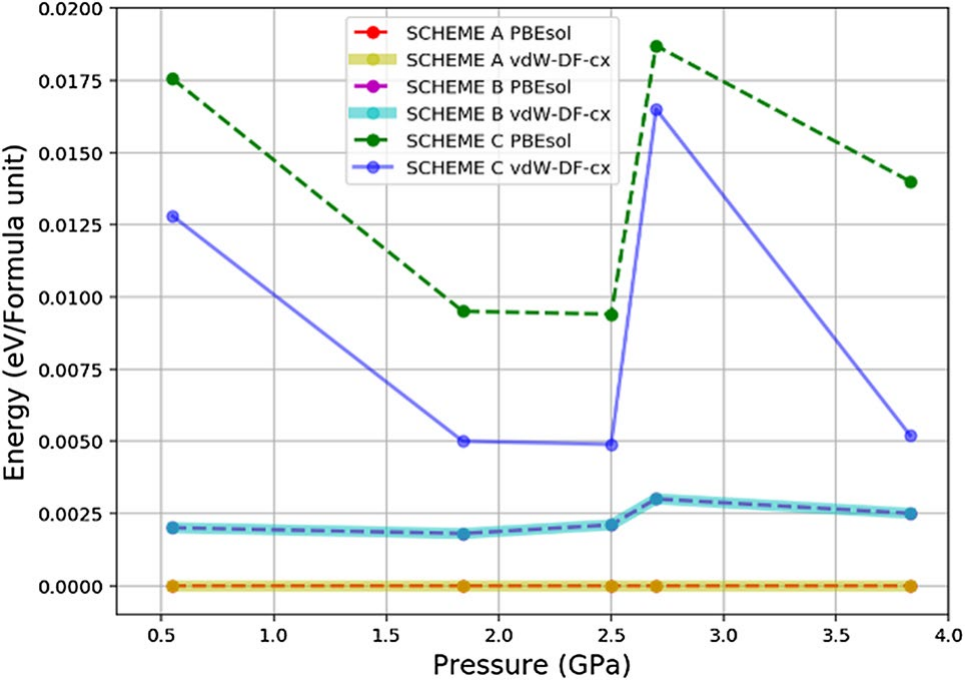
Energy of all schemes as a function of pressure. Energy of SCHEME A is selected as a reference.

The difference between Schemes A and C though is the MA cation orientations in spherical voids, MA cations point to [111] and [$\bar{1} \bar{1} \bar{1}$] directions in the latter. In the absence of pressure, the corresponding unrelaxed total energies are of the relationship $\hbox {A}< \hbox {B} < \hbox {C}$ (see also Fig. 5).

We further performed a series of structural optimisation starting with Scheme C in which, even though the net dipole moment is zero, the MA cations of $[S]_1$ and $[S]_2$ are, in fact, in an unstable equilibrium. They have the potential to evolve into any lower energy configuration. The system of Scheme C was fully optimised by relaxing atoms and cells under a discretised set of pressures in accordance with the experimentally reported results^37^, i.e., 0.55, 1.84, 2.5, 2.7, and 3.83 GPa. As a result, reported in Fig. 4, we discovered that at low pressures, 0.55 (b) and 1.84 GPa (c), the MA molecules in the spherical voids rearrange themselves into new configurations in which the net dipole is finite but not very large compared with that of Scheme B. Specifically, an MA cation in $[S]_1$ remains directionally unchanged as well as the other, while the one in $[S]_2$ reorients its dipole towards *b*-axis, resulting in a finite dipole moment at 0.55 GPa. At higher pressure, however, the relaxed structure resembles that of Scheme A where the net dipole vanishes and the MA molecules in spherical voids prefer aligning with a crystal plane, but when it reaches 3.83 GPa the relaxed Scheme C adopts non-zero polarisation with an MA cation in $[S]_1$ being slightly off-centre. Moreover, the scheme is relatively energetically stable during the pressure range 1.8–2.5 GPa as demonstrated by calculations of both PBEsol and vdW-DF-cx, which are plotted in Fig. 5.

It is worth noting that, though we did not constrain the position of any atoms in the simulation cell, whenever the structure adjusts to the equilibrium configuration, the MA molecules in the dumbbell-like voids do not realign themselves whatsoever. Instead they remain parallel to the voids in the direction we initially place them. Thus, in MAPI crystals, the $Pb{-}I$ inorganic framework has a tendency to impose a partial constraint on the organic cations.

We also performed structural optimisation starting from Scheme B, which initially has maximum net dipole, under pressure between 0.55 and 3.8 GPa. At low pressure, the MA molecules in spherical voids deflect slightly from their original positions. They tend to avoid aligning in the same direction to their neighbouring molecules. The structure, despite providing highest dipole moment, results in moderate energy states of which are more than Scheme A but much lower than Scheme C, thereby giving rise to a couple of nearly invariant energy profiles in Fig. 5. At 3.8 GPa, where the cell is subjected to higher pressures, the MA molecules in the spherical voids and its *c*-axis neighbours deflect and form a scissoring pair. Notably, this is the only case where the MA molecules in dumbbell-like voids deflected away from the voids in which they reside. While the MA molecules in the spherical voids alter their positions according to the corresponding shrinkage, the MA cation in dumbbell-like void that experiences enough repulsion from another MA also acts against the $Pb{-}I$ frameworks by distorting them.

As previously mentioned, the locking of organic molecules in HOIPs has been speculated in both experiment and in ab initio molecular dynamics studies^55,56^. Experimental studies have observed that HOIPs with large organic cations, e.g. formamidimium (FA), have longer lifetime when doped with smaller cations. One explanation is that doping with smaller cations distorts the inorganic $Pb{-}I$ network. The distorted $Pb{-}I$ network, then, constrains the movement of the larger organic cations. In the case of FA molecule, the rotation becomes hindered due to the bent angle at the centre of the molecule^56^. However, Ghosh *et al.*^56^ have no report on the orientation of MA molecule. As MA molecules are smaller and have stick-like shape with no bent angles, their rotation would be less affected by the shrinkage of the void.

As pressure is applied to MAPI, the embedded molecular voids contract, inadvertently leading to stronger vdW interaction between *H* atoms and *I* atoms. MA’s on-axis rotation is likely to enhance $I_2$ formation, shown in our previous work^9^. As MA is locked into its position and unable to spin freely at high pressure, it will eventually result in a faster degradation, since Pb–I bond strength tends to scale up with pressure, while the vdW interaction between *I* and *H* in MA has an inverse relationship.

## Conclusion

Effects of the organic molecular orientations and dipolar orientational configurations of methylammonium (MA) cations on $MAPbI_3$ were systematically and thoroughly investigated with the help of DFT. We found that by applying rigid flips to the MA cation, the energy barriers calculated from different incorporated vdW display different energy profiles and crystal structures. Three delegating dipolar configurations are suggested and evaluated using vdW-DF-cx under pressures. Scheme A remains the lowest energy structure despite being optimised under pressures, and more interestingly vdW-DF-cx and PBEsol give the same amount of energy. On the contrary, the energy profiles under pressure are solidly shifted from each other in Scheme C, even though it possesses zero polarisation: the vdW interaction seems to emphasise the relatively more stable energy. Despite being the highest polarised configuration, the energy profiles accounting for both PBEsol and vdW-DF-cx in Scheme B take second place.

## Methods

In this research, total energy and other physical properties of cubic supercell of MAPI were thoroughly investigated using Quantum Espresso Package^57^. We used Ultrasoft pseudopotentials to describe the core and valence electrons imposed in a plane wave basis set^58^. For 8-formula-unit simulation cells ($2\times 2\times 2$ supercell of cubic phase I), the energy cutoff was tested to be 90 Ry (1224.5 eV) as well as the unshifted *k*-point mesh gridded into $6\times 6\times 6$ by Monkhorst–Pack scheme^59^, satisfying the convergence threshold of 2 meV (0.021 meV/atom). This enables us to examine in detail the difference between each MA molecule orientation precisely. For the exchange-correlation potential, we have used a generalised gradient approximation (GGA) developed by Perdew–Burke–Ernzerhof (PBE)^60^. However, there are a number of physical phenomena that need special treatments, such as relativistic effects of *Pb* atoms and van der Waals (vdW) interaction between *H* and *I* atoms. The relativistic effects are incorporated in the PBEsol functional^61^, which we studied both scalar and fully relativistic effects. The vdW interaction is incorporated in the rVV10^41^, vdW-DF^42,43^, vdW-DF2^44^, vdW-DF2-b86r^45,46^, vdW-DF-cx^47^, vdW-DF-ob86^48^ functionals. We used the cubic phase as a benchmark since it is the simplest phase where the simulation cell contains only one formula unit of $MAPbI_3$.

As for the dipolar configurations, the MA molecules are depicted by arrows of which *N* is the head and *C* the tail. Among a large number of possibilities of aligning the MA cations, we chose to investigate a number of extreme cases where (1) the MA orientation gives the maximum dipole moment (Scheme B) and (2) two other cases where the total dipole moment is zero (Scheme A and Scheme C). The schematic models of each simulation cells are shown in Fig. 3. Scheme B is the configuration in which the MA orientations give up the centrosymmetric property^62^ and results in the maximum net dipole moment. Schemes A and C give the perfect centrosymmetric property, with their net dipole moment vanish. The difference between Schemes A and C is the MA orientations in the spherical voids. The latter does not allow the dipoles to align along simulation cell basis vectors, but to point along direction $\langle 111\rangle$, as discussed in the dipolar configurations under pressures section.

## Supplementary information

Supplementary Informations.

## References

[1] Zhou H (2014). Interface engineering of highly efficient perovskite solar cells. Science.

[2] Brenner TM, Egger DA, Kronik L, Hodes G, Cahen D (2016). Hybrid organic–inorganic perovskites: Low-cost semiconductors with intriguing charge-transport properties. Nat. Rev. Mater..

[3] Green MA, Ho-Baillie A, Snaith HJ (2014). The emergence of perovskite solar cells. Nat. Photon..

[4] Berry J (2015). Hybrid organic-inorganic perovskites (hoips): Opportunities and challenges. Adv. Mater..

[5] Mitzi DB, Feild C, Harrison W, Guloy A (1994). Conducting tin halides with a layered organic-based perovskite structure. Nature.

[6] Frost JM (2014). Atomistic origins of high-performance in hybrid halide perovskite solar cells. Nano Lett..

[7] Weller MT, Weber OJ, Henry PF, Di Pumpo AM, Hansen TC (2015). Complete structure and cation orientation in the perovskite photovoltaic methylammonium lead iodide between 100 and 352 k. Chem. Commun..

[8] Bechtel JS, Seshadri R, Van der Ven A (2016). Energy landscape of molecular motion in cubic methylammonium lead iodide from first-principles. J. Phys. Chem. C.

[9] Klinkla R, Sakulsupich V, Pakornchote T, Pinsook U, Bovornratanaraks T (2018). The crucial role of density functional nonlocality and on-axis ch 3 nh 3 rotation induced i 2 formation in hybrid organic-inorganic CH3NH3 pbi 3 cubic perovskite. Sci. Rep..

[10] Sukmas W (2019). Organic molecule orientations and Rashba–Dresselhaus effect in $$\alpha $$-formamidinium lead iodide. J. Phys. Chem. C.

[11] Yang Y, You J (2017). Make perovskite solar cells stable. Nature.

[12] Saliba M (2016). Incorporation of rubidium cations into perovskite solar cells improves photovoltaic performance. Science.

[13] Yang WS (2015). High-performance photovoltaic perovskite layers fabricated through intramolecular exchange. Science.

[14] Yang WS (2017). Iodide management in formamidinium-lead-halide-based perovskite layers for efficient solar cells. Science.

[15] McMeekin DP (2016). A mixed-cation lead mixed-halide perovskite absorber for tandem solar cells. Science.

[16] Correa-Baena J-P (2017). The rapid evolution of highly efficient perovskite solar cells. Energy Environ. Sci..

[17] Manser JS, Saidaminov MI, Christians JA, Bakr OM, Kamat PV (2016). Making and breaking of lead halide perovskites. Acc. Chem. Res..

[18] Shahbazi M, Wang H (2016). Progress in research on the stability of organometal perovskite solar cells. Sol. Energy.

[19] Pathak S (2015). Atmospheric influence upon crystallization and electronic disorder and its impact on the photophysical properties of organic–inorganic perovskite solar cells. ACS Nano.

[20] Zhang F (2017). Perovskite CH3NH3PbI3−xBrx single crystals with charge-carrier lifetimes exceeding 260 $$\mu $$s. ACS Appl. Mater. Interfaces.

[21] Grancini G (2017). One-year stable perovskite solar cells by 2D/3D interface engineering. Nat. Commun..

[22] Wang Y (2013). Density functional theory analysis of structural and electronic properties of orthorhombic perovskite CH3NH3PbI3. Phys. Chem. Chem. Phys..

[23] Egger DA, Kronik L (2014). Role of dispersive interactions in determining structural properties of organic-inorganic halide perovskites: Insights from first-principles calculations. J. Phys. Chem. Lett..

[24] Lee JH, Lee J-H, Kong E-H, Jang HM (2016). The nature of hydrogen-bonding interaction in the prototypic hybrid halide perovskite, tetragonal CH3NH3PbI3. Sci. Rep..

[25] El-Mellouhi F (2016). Hydrogen bonding and stability of hybrid organic–inorganic perovskites. ChemSusChem.

[26] Saliba M (2016). Cesium-containing triple cation perovskite solar cells: Improved stability, reproducibility and high efficiency. Energy Environ. Sci..

[27] Yun JS (2016). Critical role of grain boundaries for ion migration in formamidinium and methylammonium lead halide perovskite solar cells. Adv. Energy Mater..

[28] Sakulsupich, V. Theoretical studies of electronic properties of Methylammonium lead iodide perovskite under high pressure. Ph.D. thesis, Chulalongkorn University (2016).

[29] Shockley W, Queisser HJ (1961). Detailed balance limit of efficiency of p–n junction solar cells. J. Appl. Phys..

[30] Knutson JL, Martin JD, Mitzi DB (2005). Tuning the band gap in hybrid tin iodide perovskite semiconductors using structural templating. Inorg. Chem..

[31] Brivio F, Walker AB, Walsh A (2013). Structural and electronic properties of hybrid perovskites for high-efficiency thin-film photovoltaics from first-principles. APL Mater..

[32] Amat A (2014). Cation-induced band-gap tuning in organohalide perovskites: Interplay of spin-orbit coupling and octahedra tilting. Nano Lett..

[33] Kumawat NK, Tripathi MN, Waghmare U, Kabra D (2016). Structural, optical, and electronic properties of wide bandgap perovskites: Experimental and theoretical investigations. J. Phys. Chem. A.

[34] Wang Y (2015). Pressure-induced phase transformation, reversible amorphization, and anomalous visible light response in organolead bromide perovskite. J. Am. Chem. Soc..

[35] Jiang S (2016). Pressure-dependent polymorphism and band-gap tuning of methylammonium lead iodide perovskite. Angew. Chem. Int. Ed..

[36] Ong KP, Goh TW, Xu Q, Huan A (2015). Structural evolution in methylammonium lead iodide CH3NH3PbI3. J. Phys. Chem. A.

[37] Szafranski M, Katrusiak A (2016). Mechanism of pressure-induced phase transitions, amorphization, and absorption-edge shift in photovoltaic methylammonium lead iodide. J. Phys. Chem. Lett..

[38] Knop O, Wasylishen RE, White MA, Cameron TS, Oort MJV (1990). Alkylammonium lead halides. Part 2. ch3nh3pbx3 (x= cl, br, i) perovskites: Cuboctahedral halide cages with isotropic cation reorientation. Can. J. Chem..

[39] Chen T (2015). Rotational dynamics of organic cations in the CH3NH3PbI3 perovskite. Phys. Chem. Chem. Phys..

[40] Li J, Rinke P (2016). Atomic structure of metal-halide perovskites from first principles: The chicken-and-egg paradox of the organic–inorganic interaction. Phys. Rev. B.

[41] Sabatini R, Gorni T, De Gironcoli S (2013). Nonlocal van der Waals density functional made simple and efficient. Phys. Rev. B.

[42] Dion M, Rydberg H, Schröder E, Langreth DC, Lundqvist BI (2004). Van der Waals density functional for general geometries. Phys. Rev. Lett..

[43] Thonhauser T (2015). Spin signature of nonlocal correlation binding in metal–organic frameworks. Phys. Rev. Lett..

[44] Lee K, Murray ÉD, Kong L, Lundqvist BI, Langreth DC (2010). Higher-accuracy van der Waals density functional. Phys. Rev. B.

[45] Hamada I (2014). van der Waals density functional made accurate. Phys. Rev. B.

[46] Hamada, I. Erratum: van der Waals density functional made accurate [Phys. Rev. B 89, 121103 (r)(2014)]. Phys. Rev. B **91**, 119902 (2015).

[47] Berland K, Hyldgaard P (2014). Exchange functional that tests the robustness of the plasmon description of the van der Waals density functional. Phys. Rev. B.

[48] Klimeš J, Bowler DR, Michaelides A. (2011). Van der Waals density functionals applied to solids. Phys. Rev. B.

[49] Vitos L, Ruban A, Skriver HL, Kollar J (1998). The surface energy of metals. Surf. Sci..

[50] Ropo M, Kokko K, Vitos L (2008). Assessing the perdew-burke-ernzerhof exchange-correlation density functional revised for metallic bulk and surface systems. Phys. Rev. B.

[51] Tao J, Perdew JP, Staroverov VN, Scuseria GE (2003). Climbing the density functional ladder: Nonempirical meta-generalized gradient approximation designed for molecules and solids. Phys. Rev. Lett..

[52] Langreth, D. C. & Vosko, S. Response functions and nonlocal approximations. In Advances in quantum chemistry, vol. 21, pp.175–199 (Elsevier, 1990).

[53] Elliott P, Burke K (2009). Non-empirical derivation of the parameter in the b88 exchange functional. Can. J. Chem..

[54] Vydrov OA, Van Voorhis T (2010). Nonlocal van der waals density functional: The simpler the better. J. Chem. Phys..

[55] Capitani F (2017). Locking of methylammonium by pressure-enhanced h-bonding in (CH3NH3) pbbr3 hybrid perovskite. J. Phys. Chem. C.

[56] Ghosh D, Walsh Atkins P, Islam MS, Walker AB, Eames C (2017). Good vibrations: Locking of octahedral tilting in mixed-cation iodide perovskites for solar cells. ACS Energy Lett..

[57] Giannozzi P (2009). Quantum espresso: A modular and open-source software project for quantum simulations of materials. J. Phys.: Condens. Matter.

[58] Vanderbilt D (1990). Soft self-consistent pseudopotentials in a generalized eigenvalue formalism. Phys. Rev. B.

[59] Monkhorst HJ, Pack JD (1976). Special points for brillouin-zone integrations. Phys. Rev. B.

[60] Perdew JP, Burke K, Ernzerhof M (1996). Generalized gradient approximation made simple. Phys. Rev. Lett..

[61] Perdew JP (2008). Restoring the density-gradient expansion for exchange in solids and surfaces. Phys. Rev. Lett..

[62] Govinda S (2016). IS CH 3 NH 3 PBI 3 polar. J. Phys. Chem. Lett.

