# Supplementary material for: Preferred oriented cation configurations in high pressure phases IV and V of methylammonium lead iodide perovskite
Source: Sci Rep. 2020 Dec 3;10:21138. doi: 10.1038/s41598-020-77852-y (PMC7713306; doi:10.1038/s41598-020-77852-y)
Supplement: Supplementary file 1 — Supplementary Informations. [file 41598_2020_77852_MOESM1_ESM.pdf]

Supplementary Material

Preferred Oriented Cation Configurations in High Pressure Phases IV and V of Methylammonium Lead Iodide Perovskite

Wiwittawin Sukmas,[1,2] Vichawan Sakulsupich,[1,2] Prutthipong Tsuppayakorn-aek,[1,2] Udomsilp Pinsook,[1,2] Teerachote Pakornchote,[1,2] Rakchat Klinkla,[1,2] and Thiti Bovornratanaraks[1,2,*]

[1] *Extreme Conditions Physics Research Laboratory (ECPRL) and Physics of Energy Materials Research Unit (PEMRU), Department of Physics, Faculty of Science, Chulalongkorn University, 10330 Bangkok, Thailand*

[2] *Thailand Center of Excellence in Physics, Ministry of Higher Education, Science, Research and Innovation, 328 Si Ayutthaya Road, Bangkok 10400, Thailand*

(Dated: October 2, 2020)

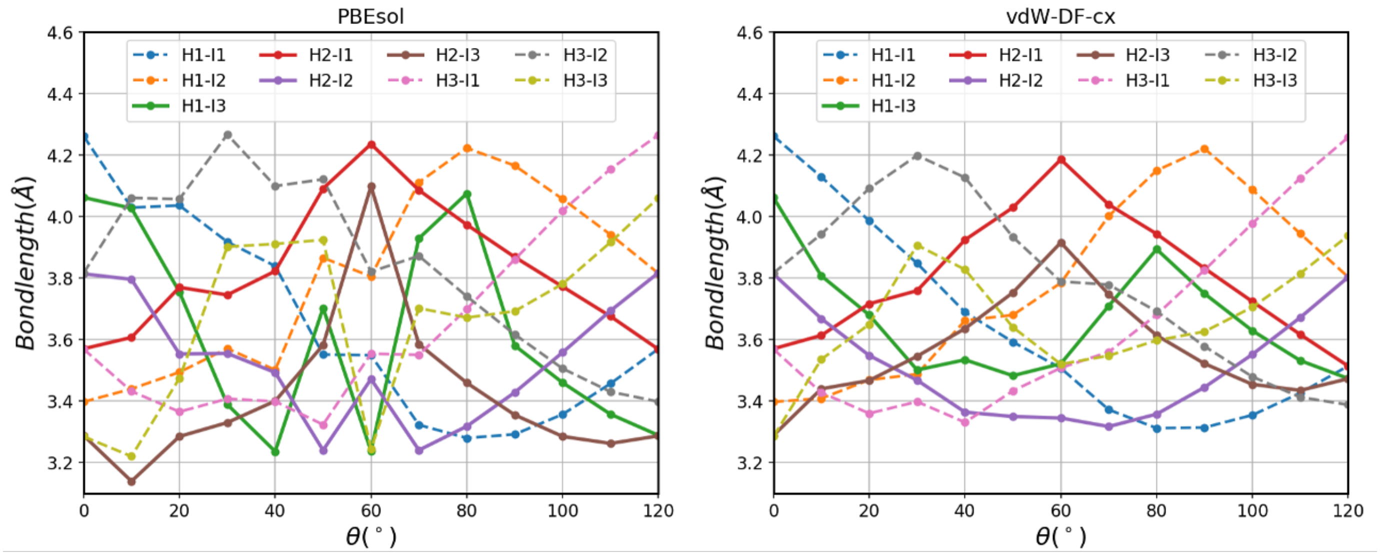

FIG. S1: Various pairs of bond lengths calculated in PBEsol and vdW-df-cx schemes, respectively.

H-I BOND LENGTHS

Figure S1 reports nine pairs of H-I bond lengths evaluated by two schemes, i.e., PBEsol and vdW-DF-cx. It is well known that PBEsol explicitly disregards the dispersive interaction of $H - I$ pairs that eventually entails a set of comparatively larger distances. Overall, the bond lengths in vdW-DF-cx scheme are significantly shortened as can clearly be seen, for example, at some apices at 60° corresponding to $H2 - I1$, $H2 - I3$, and $H2 - I2$–these peaks are eroding. Interestingly, the $H1 - I3$ profiles behave differently in both schemes, that is when dispersive interaction is taken into consideration the profile becomes smoother especially in the vicinity of $40° - 80°$.

PHASE TRANSITION UNDER PRESSURE

Increasing pressure results in a series of phase transitions. We calculated the enthalpies corresponding to the cubic phase, high pressure phase IV, and high pressure phase V at different pressures. The starting atomic positions of phases IV and V are taken from Szafranski [1]. All atoms are allowed to be fully relaxed and adjusted to confirm the possible lowest energy. The system's enthalpy as a function of pressure between 0 and 100 GPa was interpolated by the expression of 3rd-order Birch–Murnaghan equations of states [2], which is expressed as,

$$E(V) = E_0 + \frac{9V_0B_0}{16}\left(\left[\left(\frac{V_0}{V}\right)^{\frac{2}{3}} - 1\right]^3 B_0' + \left[\left(\frac{V_0}{V}\right)^{\frac{2}{3}} - 1\right]^2 \left[6 - 4\left(\frac{V_0}{V}\right)^{\frac{2}{3}}\right]\right). \tag{1}$$

The plots between the calculated enthalpy difference (ΔH) versus pressure corresponding to three phases of MAPI are reported in figure S2. The inset displays the zoomed in version of the plot indicating a small region of two phase transitions. According to our calculations, MAPI adopts a tetragonal structure (Phase II) at ambient pressure, consistent with previously experimental reports [3, 4], while it undergoes a phase transition to Phase IV at 0.3 GPa. Finally, Phase IV morphs into Phase V at around 1.8 GPa. Compared to the previous work [1], our results are, to some extent, in a good agreement with the experimental report, though the only discrepancy at phases IV and V transition (P = 1.8 GPa instead of 2.5 GPa) is due to the underestimation stemming from the GGA functional.

Also, our result shows that at higher pressure, the inorganic cage becomes distorted. When pressure is applied, each octahedral unit retains its shape, while the angle between the octahedra and overall atomic structure deviates from Im-3 symmetry. The distortion also increases with pressure. At P = 0.55 GPa, the $Pb - I - Pb$ angle is 146.78° while the same angle reduces to 144.04° at P = 3.83 GPa. Thus pressure induces $Pb - I$ distortion but not to the degree that reconstructs the original framework, as illustrated in figure S3.

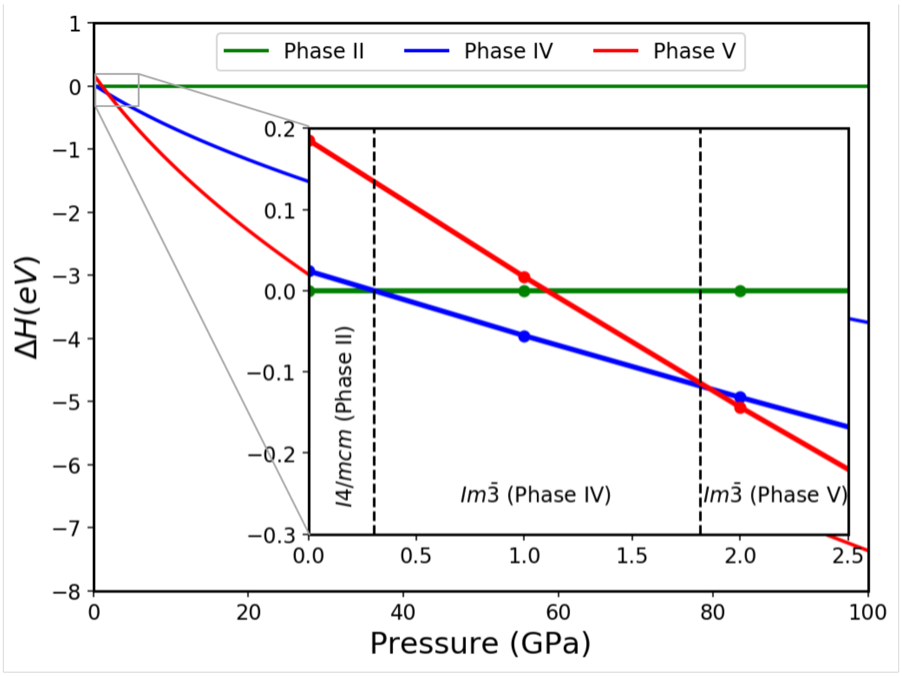

FIG. S2: The relationship between enthalpy difference (eV) and pressure (GPa) calculated by vdW-DF-cx is fitted by 3rd-order Birch–Murnaghan equations of states.

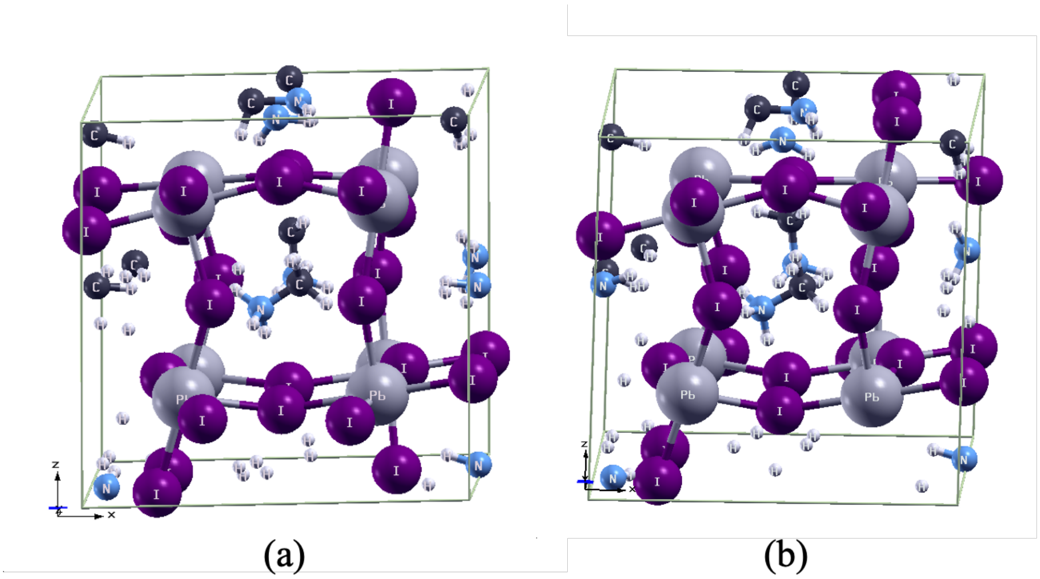

FIG. S3: Relaxed MAPI structures calculated by vdW-DF-cx. The relaxed phase IV structure at 0.55 GPa (a). The relaxed phase V structure at 3.83 GPa (b)

* Electronic address: Thiti.B@chula.ac.th

[1] M. Szafranski and A. Katrusiak, The journal of physical chemistry letters **7**, 3458 (2016).
[2] F. Birch, Physical review **71**, 809 (1947).
[3] N. Onoda-Yamamuro, T. Matsuo, and H. Suga, Journal of Physics and Chemistry of Solids **51**, 1383 (1990).
[4] M. T. Weller, O. J. Weber, P. F. Henry, A. M. Di Pumpo, and T. C. Hansen, Chemical communications **51**, 4180 (2015).
